# Effectiveness modelling of digital contact-tracing solutions for tackling the COVID-19 pandemic

**DOI:** 10.1017/S0373463321000175

**Published:** 2021-04-07

**Authors:** Viktoriia Shubina, Aleksandr Ometov, Anahid Basiri, Elena Simona Lohan

**Affiliations:** 1 Tampere University, Tampere, Finland; 2 University ‘Politehnica’ of Bucharest, Bucharest, Romania; 3 University of Glasgow, Glasgow, United Kingdom

**Keywords:** contact tracing, COVID-19, infection risk, effectiveness modelling, mobile devices, Bluetooth Low Energy (BLE), proximity detection

## Abstract

Since the beginning of the coronavirus (COVID-19) global pandemic, digital contact-tracing applications (apps) have been at the centre of attention as a digital tool to enable citizens to monitor their social distancing, which appears to be one of the leading practices for mitigating the spread of airborne infectious diseases. Many countries have been working towards developing suitable digital contact-tracing apps to allow the measurement of the physical distance between citizens and to alert them when contact with an infected individual has occurred. However, the adoption of digital contact-tracing apps has faced several challenges so far, including interoperability between mobile devices and users’ privacy concerns. There is a need to reach a trade-off between the achievable technical performance of new technology, false-positive rates, and social and behavioural factors. This paper reviews a wide range of factors and classifies them into three categories of technical, epidemiological and social ones, and incorporates these into a compact mathematical model. The paper evaluates the effectiveness of digital contact-tracing apps based on received signal strength measurements. The results highlight the limitations, potential and challenges of the adoption of digital contact-tracing apps.

## 1. Introduction and motivation

In the context of a pandemic, contact tracing is utilised as a mitigation approach to identify individuals being exposed to an infectious person, i.e. either having developed symptoms or having tested positive. Contact-tracing solutions have been seen to be highly effective to act as preventive or control measures. They can alert individuals to take actions such as self-isolation or quarantine, as discussed in Li and Guo (2020), Hernández-Orallo et al. (2020) and Troncoso et al. (2020). Contact tracing can be either digital or manual. Manual contact tracing relies on collecting the patient's contacts, e.g. by authorised personnel such as nurses, doctors and public safety forces. As manual contact tracing can be subject to human memory and several behavioural and cultural factors, as stated in Braithwaite et al. (2020) and Dropkin (2020), this paper focuses on digital contact-tracing solutions.

Although digital contact-tracing solutions are not meant to replace but rather to complement medical mitigation plans, they have been considered an effective way to stop or minimise the spread of infectious diseases in many countries. This is a particularly well-spread strategy for COVID-19 due to the high spread levels and the nature of the disease, i.e. a respiratory disease that can infect individuals who have been in close contact with an infectious person, according to Salathe et al. (2020) and Ferretti et al. (2020). With the wide availability and access to the technologies that people use for their daily activities, it has been possible to develop digital contact-tracing applications (apps) on the available devices and technologies. Broadly, consumer hand-held or wearable technology, with several miniaturised embedded sensors, can help the individuals’ devices either to measure the distance and proximity between them, and/or to be alerted if they have been in contact with a COVID-19 patient, as described in Hernández-Orallo et al. (2020), Reichert et al. (2020), Scudellari (2020), Li and Guo (2020), Rodríguez et al. (2021), Tripathy et al. (2020), Pépin et al. (2020), Stojanović et al. (2020) and Abbas and Michael (2020). Examples of sensors useful in proximity detection and user-location estimation are: chipsets, such as Global Navigation Satellite Systems (GNSS), e.g. Global Positioning System (GPS) or Galileo, Wireless Local Area Network (WLAN or Wi-Fi), Bluetooth and Bluetooth Low Energy (BLE), Ultra Wide Band (UWB), ultrasound, magnetometers, accelerometers and gyroscopes, as per the studies in Nguyen et al. (2020a,2020b) and Shubina et al. (2020a).

Owing to the urgency of finding a solution to cope with the current pandemic, the digital contact-tracing software-based apps should rely on the existing sensors on users’ devices, such as mobile phones, rather than investing in developing new infrastructure. If a solution relies on existing technologies, it can also help users to carry and use such devices, with no extra time or effort, which can maximise the app's effectiveness. The app should ideally provide a free or very low-cost, scalable, reliable, accurate and privacy-preserving solution, as explained, for example, in Li and Guo (2020), Troncoso et al. (2020) and Altuwaiyan et al. (2018).

The main goal of such proximity-detection, user-tracking or digital contact-tracing apps is to evaluate the probability of being infected, in this case with COVID-19, based on the proximity to and/or physical contact with a COVID-19-infected person. If the risk passes a certain threshold, then the digital contact-tracing app can issue an alert and can potentially advise the user to self-isolate, to take tests or to apply other preventive measures to stop the spread of the disease, as per Hernández-Orallo et al. (2020) and Li and Guo (2020). However, the adoption and the success of digital contact-tracing apps is subject to many factors, including social, personal, epidemiological and technical factors.

This paper presents a holistic outline of the existing methods, by using an interdisciplinary approach to understand the epidemiological, social and technical aspects of the digital contact-tracing solutions. This allows us to identify the current challenges towards successful future developments. In this paper, we address the following main research question: ‘How does one model, quantify and evaluate the usefulness and effectiveness of digital contact-tracing apps holistically, from the technical, social and epidemiological points of view?’

The primary contributions of this paper are as follows:

1. To offer an overview of some of the widely used wireless technologies embedded in our phones and existing wireless protocols that can be used for digital contact-tracing apps.
2. To present the technical limitations and identify technical specifications for designing digital contact-tracing apps.
3. To offer a comprehensive mathematical model based on a proposed *effectiveness metric* for the digital contact-tracing apps, defined as an estimation measure to evaluate the benefit towards disease prevention when using a digital contact-tracing app compared to the scenarios of not using a digital contact-tracing solution.
4. To discuss future perspectives, the remaining challenges to overcome and possible solutions to improve the adoption rate of the digital contact-tracing apps.

The rest of the paper is organised as follows. Section 2 reviews the related work on measuring and evaluating the effectiveness of digital contact-tracing apps. Section 3 describes the main principles of a digital contact-tracing app, with a particular focus on the typical network architectures and wireless technology chain, as well as on the decision-making process. Section 4 presents the main contribution of our work by introducing a multidimensional model to assess the effectiveness of digital contact-tracing apps. The model incorporates technological, epidemiological and social aspects. Section 5 presents a measurement-based analysis for digital contact-tracing apps, relying on received signal strength measurements (as the majority of such apps do), and Section 6 focuses on simulation-based results to compare the current state of digital contact-tracing apps across the globe. Section 7 summarises the challenges still to be tackled towards achieving higher effectiveness of digital contact tracing, as well as some ideas towards how such challenges can be overcome. Finally, the last section presents the conclusions and take-away points of our research. The Appendix provides complementary material with a brief survey of digital contact-tracing protocols and software apps existing at the time of writing this paper.

## 2. Related work

Despite the facts that there are numerous protocols developed for digital contact tracing and there are many wireless proximity-detection solutions that use wireless signals, unified mathematical models of the effectiveness of a digital contact-tracing solution are still lacking from the current literature.

A study by Hinch et al. (2020) models five scenarios of the potential of digital contact-tracing apps and states that the approach would only work in conjunction with other preventive measures, such as quarantine and social distancing.

Concerning the authors’ recent research, the survey by Shubina et al. (2020a) overviews digital contact-tracing solutions and their underlying technologies. However, this work did not address the mathematical model allowing us to analyse the effectiveness of the digital contact-tracing solution. Another recent work by Shubina et al. (2020b) presented a basic mathematical model of the maximum prevention probability of digital contact-tracing solutions. However, no further detailed models of each of the influencing factors of the application's effectiveness were given. In the present paper, we define a new effectiveness metric to measure the benefit of using a digital contact-tracing app contrasted to cases where no digital contact-tracing technology is implemented. The effectiveness metric presented here is related not only to the user adoption rates and infection risk, but also to the models on possible asymptomatic transmissions, as well as on various technical characteristics of the digital app, such as the wireless path-loss propagation model, and cloud and software reliability features.

Bonsall and Fraser (2020) discuss the advantages and limitations of contact tracing, recommending measures for achieving the so-called herd protection of the population. The study also describes another method used by China, the WeChat app, which aggregates information about their users on a central server, yet issues colour codes for the population.

Related studies to our work, i.e. the effectiveness of digital contact-tracing apps, can be classified into three main categories of studies, which discuss or propose one of the following aspects: (i) the effectiveness metrics from an epidemiological perspective, such as those by Kretzschmar et al. (2020), Rodríguez et al. (2021), Hellewell et al. (2020), Salathe et al. (2020), Ferretti et al. (2020), Hu (2020) and Hernández-Orallo et al. (2020); (ii) the effectiveness metrics that are mainly based on social aspects, including Frimpong and Helleringer (2020), Larsen et al. (2020), Almagor and Picascia (2020), Sharma et al. (2020) and Nguyen et al. (2020a), which include studies that look at user privacy and users’ behaviour, as discussed by Ajmal Azad et al. (2020); and (iii) the effectiveness metrics related to technical aspects, including the wireless signal propagation and path-loss models (Leith and Farrell, 2020b; Nguyen et al., 2020b; Spachos and Plataniotis, 2020).

Regarding the models pertaining to the epidemiological domain, Stojanović et al. (2020) presented a wearable system with sensors and a mobile phone to measure body temperature, heart rate and respiration rate in order to identify critical COVID-19 symptoms in time. Another example of digital healthcare advances was discussed by Oura (2020), where smart rings were emphasised as a promising tool for identifying early symptoms. However, no mathematical modelling regarding the ring's effectiveness was presented by Oura (2020).

From the perspective of social models, the study by Pépin et al. (2020) focused on the role of wearable devices for activity tracking during the COVID-19 pandemic, including such measures as lockdown. As a result, the researchers concluded that anonymised activity data are a valuable source of information to compare the effectiveness of different government policies for countries worldwide, adopting different levels of lockdown measures: total, partial or none.

Related research focusing on the technical domain is provided by Adib and Katabi (2013), Zhao et al. (2020) and Basiri et al. (2017). There are certain qualities that social distancing technologies must possess. The most critical aspects of a digital contact-tracing app are availability, reliability, accuracy and privacy. For better services, these apps must operate consistently and seamlessly both indoors and outdoors. For privacy-preserving purposes, they should compute the relative, rather than the absolute, position of users. Earlier research, e.g. by Basiri et al. (2017) and Mautz (2012), identified Bluetooth Low Energy (BLE) as the most apt technology for digital contact-tracing solutions due to its ubiquitous embedding in mobile phones. BLE can provide proximity-sensing signals that are seamlessly available indoors and outdoors, for free, with a relatively consistent level of accuracy in their distance-estimation solutions. Therefore, many contact-tracing apps are built upon BLE, according to Ahmed et al. (2020) and Leith and Farrell (2020a), as will also be detailed in Section 3.

BLE is used to exchange data over short distances, and this wireless technology standard has become increasingly popular since the release of the standard Bluetooth 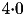 protocol. Hossain and Soh (2007) reviewed Bluetooth technology and positioning using Bluetooth technology. The decisive advantage of BLE over other existing wireless positioning technologies is that it can be deployed in several tags or beacons in different environments due to its power efficiency and low cost. The short operating range typically provides better performance than WLAN-based positioning in terms of the estimated distance/ranging error (Lohan et al., 2015). In modern devices and sensors, the effects of BLE interference can be reduced by broadcasting on widely spaced radio channels.

Still related to the technological domain, Adib and Katabi (2013) outlined a novel paradigm called Wi-Vi (also known as Wi-Fi Vision) to provide the authorities with an opportunity to track objects in specific environments and to detect potentially crowded areas. It would allow a prompt response with appropriate actions to enable social distancing, e.g. by informing people to avoid potentially crowded places. The take-away point from such research is that contact-tracing apps alone cannot mitigate the spread of disease if not followed up by concrete actions such as self-isolation and quarantine.

Other related research in terms of technological models is, for example, the study by Zhao et al. (2020), focusing on analysing the main factors affecting the received signal strength fluctuations. The authors claimed that configurations for the signal transmission power and broadcasting intervals varied for different contact-tracing apps.

Regarding the joint epidemiological and social perspectives, related works to this research can be found, for example, in Salathe et al. (2020) and Hellewell et al. (2020). Salathe et al. (2020) discussed the effectiveness of the SwissCovid digital contact-tracing app (used in Switzerland) based on empirical data. A longitudinal study made in Zurich with 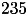 participants measured an effectiveness metric 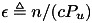. The parameters of the model used by Salathe et al. (2020) were drawn from the social and epidemiological domains: 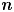 is the number of persons who got a positive result, following their voluntary tests for COVID-19 (as a consequence of an alert received through the SwissCovid app); 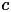 is the number of persons previously confirmed positive via a COVID-19 code; and 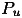 is the proportion of the Swiss population that were actual users of the SwissCovid app. The results of the study by Salathe et al. (2020) showed an effectiveness metric 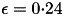. The intuition behind the ‘effectiveness metric’ defined by Salathe et al. (2020) is that, under a 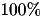 user adoption rate (i.e. 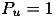), the effectiveness metric will converge to the 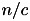 ratio, namely the number of secondary infections with respect to a certain number of index cases. Nevertheless, as discussed by Hellewell et al. (2020), the effectiveness of a contact-tracing method should be modelled not only based on the relative number of secondary infections 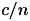 and on the user adoption rate 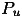, but also on the percentage of transmissions that may occur before the onset of COVID-19 symptoms, as well as on the follow-up procedures such as isolation or self-quarantine.

A broad survey on the effectiveness of social distancing measures and the use of other technological advances, written by Nguyen et al. (2020a,2020b), highlighted the joint technical and social perspectives of contact tracing. In the first part, Nguyen et al. (2020a) gave a historical overview and comparison to other disease outbreaks to classify possible strategies and to divert those lessons into recommendations. Moreover, the authors stated that localisation systems, such as Wi-Fi, cellular and GNSS, helped in many scenarios for physical distancing, monitoring of public places, contact tracing and automation. A concept of crowd detection in dynamic environments was introduced by Nguyen et al. (2020a) with the use of fingerprinting, as an approach to deal with non-line-of-sight (NLOS) propagation on the wireless signals between the user's equipment (UE) and the anchor node (AN), especially in dynamic and complicated environments (e.g. shopping mall, airport), where obstacle shadows greatly scatter the wireless signals. The second part of the survey by Nguyen et al. (2020b) was devoted to potential application scenarios of artificial intelligence to several social distancing use cases. As an outcome, the authors concluded that modern next-generation wireless system infrastructures, such as 6G, smart cities and intelligent transportation systems, could include a pandemic contact-tracing mode as part of their standard.

**Figure 1.**
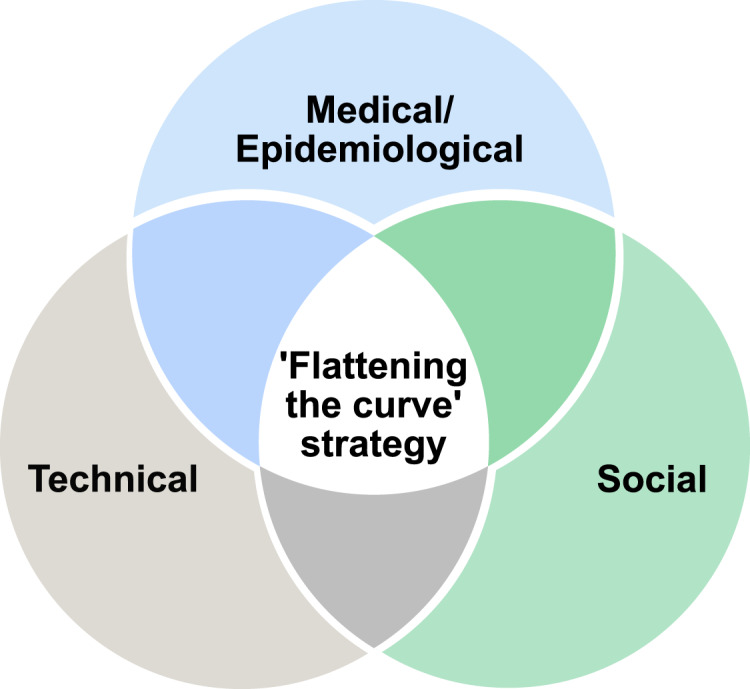
Venn diagram on the perspectives from the scope of our study

To complement the existing research mentioned above and to address the epidemiological, social and technical perspectives jointly, as illustrated in Figure 1, our paper aims at offering a combined model, which is done by analysing multidimensional aspects to achieve the best results according to the ‘flattening the curve’ strategy, as explained by Villas-Boas et al. (2020). Our multidimensional approach incorporates the epidemiological aspects (e.g. the infection risk models), the social aspects (e.g. the user adoption probabilities) and the technical aspects (e.g. the errors in estimating user proximity with a particular wireless technology, the possible outages in the cloud connectivity and the software reliability).

## 3. Principles of a digital contact-tracing app

The following subsections describe the basic principles of digital contact-tracing solutions, focusing first on the network architecture and the nodes involved in the process, and describing the technology chain from user to cloud server and back to the user.

### 3.1 Network architecture

The basic principle of a digital contact-tracing app relying on wireless signals is as follows. In the considered scenario, several users are equipped with wearables or other mobile devices distributed within a particular geographical area, e.g. a university building, a shopping mall, a restaurant, a outdoor tourist site within a city, etc. Users’ devices can monitor the wireless environment with, for example, BLE, and exchange beacons between them with a specific sampling interval.

The assumption of wireless data exchange between user devices is reasonable, as many devices, such as smartphones and smartwatches, operate upon short-range wireless communication technologies, such as BLE or Wi-Fi (Ding et al., 2020; Ometov et al., 2016). Less common short-range wireless communication technologies are ZigBee (Shao et al., 2020), typically used in industrial environments for workers and devices, and Radio Frequency Identification (RFID), as encountered in hospital environments (Ding et al., 2019). Such short-range, low-power wireless communications usually transmit some beacons with timestamps along with the users’ unique but anonymised identifiers (IDs). Therefore, any other user device equipped with a wireless receiver in the emitter range and decoding the emitted signal could sense and store the timestamped IDs of neighbouring devices. Additionally, received signal strength (RSS) measurements are typically computed and stored at the receiver side. For example, when a user transmits a BLE or Wi-Fi wireless signal, the mobile devices of the users in their neighbourhood could estimate the user-to-user distance, based on RSS, time-of-arrival (TOA) or angle-of-arrival (AOA) measurements, as discussed by Basiri et al. (2017). Once such distance is estimated, a threshold can be applied. Nowadays, a threshold of 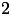 m (or about 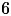 feet) is the ‘safety threshold’ adopted by many research papers focused on coronavirus infectiousness (Hernández-Orallo et al., 2020; Jeong et al., 2019; Tripathy et al., 2020). Recent studies have argued about the 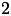 m value and found that SARS-CoV-2 can spread to more than 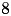 m in some industrial environments (Günther et al., 2020). In our studies, we will denote, without any loss of generality, this threshold distance as 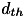, and it will be one of the model parameters. Once the receiver establishes that the distance to a neighbouring device is less than or equal to 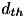, the receiver starts logging the (anonymised) IDs from the neighbouring user while the corresponding timestamps indicate that the estimated user-to-user distance is below the 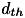 threshold. Consequently, a ledger of nearby nodes could be created per device.

The user-to-user distance can be estimated, for example, from time measurements via the wireless signal, in scenarios where available (as distance 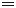 time 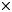 speed, and the electromagnetic signal speed is the well-known speed of light), or, more often encountered, from RSS measurements and assuming a certain path-loss propagation model. Mathematical models based on RSS will be further provided in Section 4.2. In Section 3.2, we talk about the basic scheme of a contact-tracing app and explain the stages of data processing.

### 3.2 Technology chain in a digital contact-tracing app

**Figure 2.**
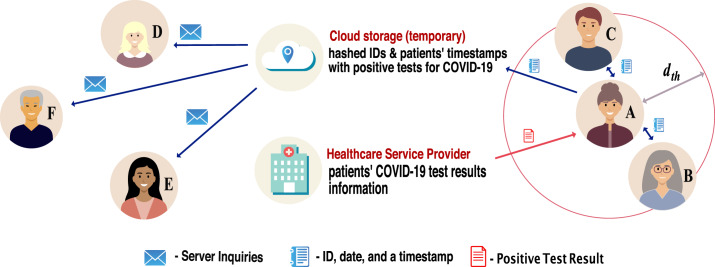
Illustration of the digital contact-tracing chain

A wireless or digital contact-tracing chain as illustrated in Figure 2 typically involves the following conceptual steps, with possible variations regarding when the actual user-to-user distance is computed and the place (user side or server side) where the computations are performed:

1. A pre-symptomatic or asymptomatic person (e.g. person A infected with SARS-CoV-2) has active contact-tracing software on his/her mobile device, and this software sends periodic beacons over the wireless channel, with a granularity defined by the software sampling rate (e.g. every 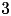 min). Such beacons contain timestamps and a user-specific ID. Typically the user-specific ID is randomly generated according to a predefined random number generator in the user device, and therefore it preserves the user's anonymity.
2. The mobile devices of all persons in the vicinity (e.g. within a radius 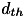) of the infectious person A, with the same mobile software enabled, receive the beacons sent by user A and measure the RSS over a predefined time duration (e.g. 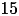 min).
3. The software converts the RSS values into user-to-user estimates by applying averaging and calibration when needed. The RSS-to-distance conversions can take place either directly on the user device or in a cloud system. In the latter case, all user devices send the RSS measurements together with the timestamps and ephemeral IDs to a cloud server.
4. If the estimated user-to-user distance is below the threshold distance 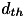, the corresponding user IDs and timestamps for the nearby users are stored for a predefined time interval (e.g. 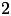 weeks) either on the user device (in a decentralised approach, see also Section 3.3) or on the cloud server (in a centralised approach).
5. If user A never gets tested, nothing happens with the information mentioned above. However, if user A gets tested and gets a positive COVID-19 result, the positive-test information is transmitted to the cloud server, either automatically from the authorised health service provider or with the user's help. In the latter case, the user can also choose whether or not to send the information about a positive result to the server.
6. The cloud server informs all user devices that have the mobile app enabled about the ephemeral IDs and the timestamps of the user who has tested positive. These IDs and timestamps pertain only to a certain predefined time window, which is set to be the infectious period window (e.g. from a few days before the onset of symptoms or before the positive test if no symptoms and until the day of the positive test).
7. In a centralised approach (see also Section 3.3), the server also sends an exposure notification signal to all users deemed to have been in the vicinity of the infectious user during the infectious period.
8. In a decentralised approach (see also Section 3.3), each user device computes on its own the probability to have been infected, based on the pre-stored ephemeral IDs and timestamps.

Clearly, each of these steps has to cope with several sources of errors from the technology point of view, namely:

- Low granularity of wireless beacons, i.e. no signalling between users within a large time window due, for example, to the BLE signal being off on the mobile device, the phone battery being depleted, or wireless connectivity errors;
- Wireless channel impairments, such as large fluctuations in RSS values due to reflections, refractions, diffractions or scatterings in the waveform path, or obstacles in the signal path, such as walls, shelves, etc., which affect the accuracy of the user-to-user distance estimates;
- Timing and other synchronisation errors, such as incorrectly stored timestamps, which can affect the estimates of the exposure duration;
- Cloud connectivity errors, such as the cloud server being down or tampered with or long-range wireless connectivity unavailable; and
- Other software-related errors.

### 3.3 Centralised versus decentralised decision-making approaches

As surveyed in our previous study (Shubina et al., 2020b), two concepts describe the main contact-tracing decision-making approaches:

- Centralised approach. This indicates that primary information – i.e. the temporary IDs and timestamps of active users – is aggregated on a central server. In this scenario, the server holds the information about the downloads and active devices.
- Decentralised approach. This means that the information is distributed among the involved entities. Therefore, the users store relevant information locally on their devices. This information includes their own temporary IDs, the temporary IDs of the nearby users and timestamps. Devices communicate with the server solely to state COVID-19 symptoms or to download the temporary IDs of other users who have registered an infection to juxtapose with the local database.

In centralised decision-making approaches, risk analysis is performed at the server side. Therefore, after calculating the probability 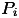 of a user to become infected, the server notifies each user about the results. On the contrary, in a decentralised approach, 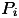 is calculated by each device. In this case, the server follows the data minimization principle. It possesses a lower amount of user-related information than in the centralised approach, and no data about the contacts who crossed their paths need to be broadcast.

Detailed discussions about the advantages and possible shortcomings of a decentralised decision-making process versus a centralised one can be found, for example, in Shubina et al. (2020a) and references therein.

### 3.4 An example from UK contract-tracing app

**Figure 3.**
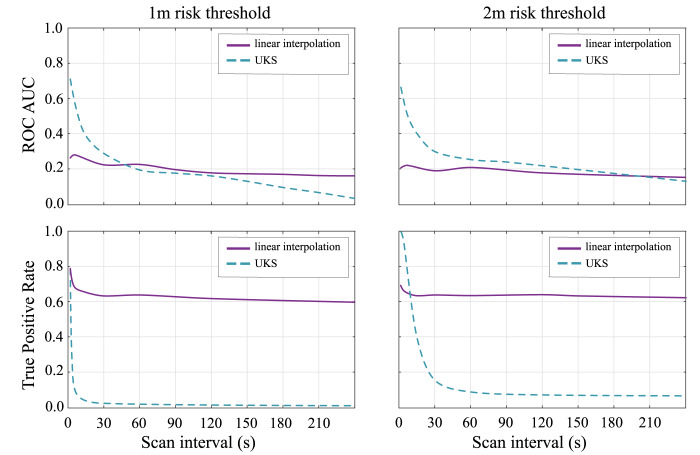
The ROC AUC for both the UKS and linear interpolation models to compare UKS performance across 4000 random walks with varying scanning interval

After the previous general discussions on network architectures (Section 3.1), the principles of a digital contact-tracing app (Section 3.2 and the decision-making processes (Section 3.3), this present section provides a concrete example based on the UK contact-tracing app, called NHSX. NHSX uses BLE measurements, considered at the app-defined scanning intervals, and decides about the infection risk of users in close proximity to each other by integrating past and present measurements through an unscented Kalman smoother (UKS) (Lovett et al., 2020). Compared with the traditional linear interpolation, UKS is a compromise between computational complexity, mathematical complexity and accuracy. For example, based on our simulations on the MIT dataset (MIT Matrix Data, 2020), we understand that the performance accuracy of such an app declines with respect to the scanning interval, namely the interval at which the BLE scans are performed. The performance metric is defined here as the ability to correctly classify the infection risk under two different proposed distance thresholds 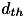 (either 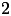 m distance or 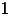 m). The performance metrics provided as an example in Figure 3 are the receiver operation characteristic (ROC) area under curve (AUC) and the true positive rates (or detection probability 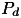). The ROC is defined as the detection probability 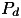 versus false-alarm rate 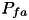, and the AUC computes the area below the 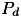 versus 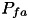 curves (more mathematical details on 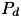 and 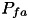 are given in Section 4). A higher ROC AUC and a higher 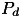 indicate better performance results than with a low ROC AUC and low true positive rates, respectively.

As shown in Figure 3, UKS strongly outperforms the linear interpolation model at scan intervals below 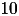 s; however, this performance improvement is minor at scan intervals of 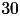 s or higher and disappears above 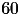 s scanning. It is far below the scan intervals of an average 3–4 min, which have been proposed by Google and Apple initially. The scan interval issue has been addressed in the latest release of the Google and Apple Exposure Notification (GAEN) Application Programming Interface (API), though. It is also important to emphasise that the higher sampling rate that guarantees the out-performance of the UKS versus linear interpolation may result in higher power consumption. However, this performance improvement is minor at scan intervals of 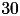 s or higher and disappears entirely above 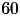 s scanning. The 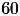 s scanning is far below the scan intervals of an average 3–4 min which had been initially proposed by Google and Apple for a fair tradeoff with battery consumption on the mobile. Leith and Farrell (2020c) noted that the universal benchmark proposed by Google and Apple only scans each 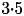
–5 min. It raises the question of whether advanced integration methods such as the UKS can improve the contact-tracing system in practice.

In conclusion, there are still several challenges in designing the technical parameters of a contact-tracing app, such as the BLE scanning interval, the choice of the algorithm to integrate BLE measurements and take a decision about the infection risk, as well as the safety or risk threshold 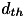. Some of these parameters, such as the safety or risk threshold 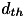, will also be a part of our multidimensional model, described next in Section 4, while the others, such as the integration algorithm and scanning interval, will not be modelled explicitly, but will be a part of the additional noise in the model explained in Section 4.1. More discussions about the tradeoffs and open challenges in digital contact-tracing apps are offered in Section 7.

## 4. Proposed multidimensional modelling

**Figure 4.**
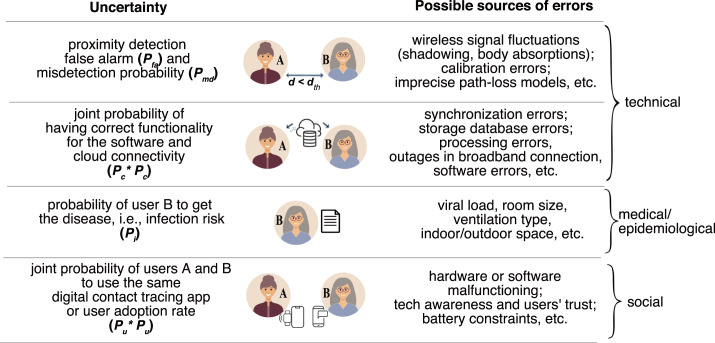
Illustration of the basic steps and associated probabilities of a wireless COVID-19 contact-tracing application

Following the diagram in Figure 4 and our previous work (Shubina et al., 2020b), we propose the effectiveness metric 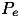 to measure the utility of a digital contact-tracing solution, expressed as 1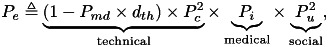 where

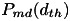
 is the so-called misdetection probability, meaning the probability to estimate the user-to-user distance as being above the considered threshold 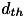 (above 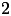 m) based on the observed RSS values when in fact it was below the threshold (a model for 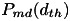 is derived in Section 4.2);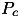 is the probability that the user device is reliably connected to the cloud server, and that the contact-tracing software is working properly, and therefore it is a measure of the connectivity and software reliability (it will be further detailed in Section 4.3);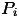 is the infection risk, i.e. the tangible probability that user B becomes infected in case they crossed paths with user A who has tested positive for COVID-19 (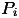 will be further detailed in Section 4.4); and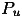 is an average probability that a user utilises the considered digital contact-tracing app, i.e. also measured as the user adoption rate (it will be further detailed in Section 4.5).

Note that Equation (1) is further explained by the following assumptions:

1. The joint probability of two devices detected to be in close proximity to each other and to use the same contact-tracing app, assuming independent users, is the product of the individual probabilities, namely 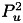.
2. The threshold distance 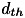 currently adopted by most apps is 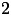 m, and it represents the maximum distance between an infectious and a non-infectious user, which creates an exposure alert if the two users under consideration stayed in each other's proximity for at least 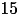 min (Jeong et al., 2019; Hernández-Orallo et al., 2020; Tripathy et al., 2020).
3. The so-called false-alarm probability 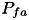 (or the false-positive rate), which is not a direct part of the model in Equation (1), shows the probability of incorrectly estimating the user-to-user distance as being below the considered safety threshold 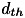. Admittedly, 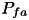 does not impact the prevention probability or effectiveness of a digital contact-tracing app, where non-infectious users are erroneously identified as infectious users. It is a potential research direction to determine whether 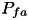 affects the population perception of the contact tracing usefulness as a reliable approach. High levels of 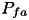 would mean that some non-infectious users would be recommended to self-isolate without actual need. Therefore, higher levels of 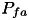 could affect the adoption rate of contact-tracing solutions (i.e. fewer users would be willing to install an app with high 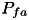) and therefore could indirectly influence the 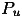 levels.

We remark that the effectiveness probability 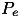 shown in Equation (1) is the effectiveness assuming all users of a digital contact-tracing app cooperate with follow-up regulations obeying the recommended measures such as a self-quarantine after an exposure alert.

Examples relying on our model and based on simulated data are given in Section 6. The next subsections will shed more details on modelling the different probabilities in Equation (1).

We will focus on BLE signal models, as BLE is by far the most used wireless signal in contact-tracing apps nowadays, as shown in Section 5. Nevertheless, except for the numerical values for the parameters used in BLE path-loss modelling, the proposed models below hold for any wireless signals that can be used for RSS-based distance estimation, e.g. Wi-Fi, RFID, etc.

### 4.1 Modelling RSS fluctuations based on BLE measurements on mobile phones

In order to derive a model for the misdetection and false-alarm probabilities, we start first by summarising the different RSS models for BLE signal propagation. We remark that the misdetection probabilities are also referred to in the literature as false-negative detection rates (i.e. a user is incorrectly found not to be exposed), while the false-alarm probabilities are also referred to in the literature as false-positive detection rates (i.e. a user is incorrectly found to be exposed). Both false-negative 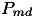 and false-positive 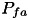 indicators are hurtful to the effectiveness of an app. The 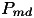 plays a direct role in Equation (1), as any undetected user that has been exposed to the virus can spread the virus further and thus diminish the effectiveness of the contact-tracing app. The false positives reflected in 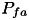 play an indirect role by influencing the user adoption rate 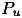, because a high 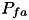 means that many users, following a false alert, are sent unnecessarily into quarantine, and therefore, in time, the perceived usefulness of such an app decreases and users’ adoption rate 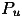 decreases.

In what follows, we will denote by 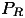 the received signal power/RSS, expressed in dBm (decibels referenced to 1 mW), by 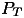 the transmitted signal (in dBm) at 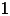 m away from the transmitter, and by 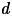 the user-to-user distance (i.e. the distance between transmitter and receiver).

Two of the most commonly encountered RSS models in the literature are the Gaussian single-slope model for RSS values expressed in dBm, as stated by Lohan et al. (2015), Clark et al. (2020) and Leith and Farrell (2020b), and the log-normal model for RSS values in linear scale, according to Lovett et al. (2020).

The Gaussian single-slope model shows the dependence of the 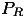 on the distance 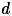 via
2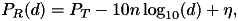 where 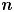 is a path-loss coefficient specific to the considered environment (e.g. indoor, train, outdoor, etc.) and 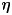 is a Gaussian-distributed random variable of zero mean and 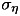 standard deviation. Values for 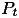, 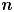 and 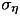 have already been studied in various environments, based on BLE measurements for contact-tracing applications. Six examples can be found in Table 1.

**Table 1.** RSS path-loss parameters based on measurements with BLE signals

Model					
index	Reference	Measured scenario	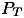 (dBm)	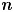 (–)	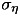 (dB)
M1	Clark et al. (2020), Leith and Farrell (2020b)	Indoors, meeting room	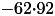	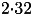	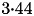
M2	Clark et al. (2020), Leith and Farrell (2020b)	Indoors, inside a train	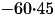	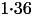	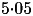
M3	Clark et al. (2020), Leith and Farrell (2020b)	Outdoors, Dublin city streets	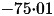	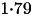	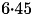
M4	Lohan et al. (2015)	Indoors, university corridors	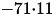	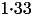	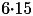
M5	Röbesaat et al. (2017)	Indoors, office room	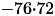	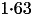	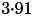
M6	Proposed linear-regression fit on MIT-Matrix data	Indoors, MIT (MIT Matrix Data, 2020)	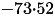	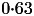	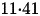

An equivalent, but less-often used, RSS path-loss model is the log-normal model for RSS values in linear scale 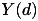 (Lovett et al., 2020), where 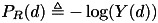, and 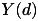 is modelled as a log-normaldistributed variable of mean 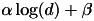 and variance 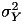. Lovett et al. (2020) analysed the MIT-Matrix data provided by MIT Matrix Data (2020) through a gradient boosting regressor and found the best-fit parameters 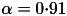, 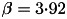 and 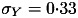.

### 4.2 Modelling the misdetection and false-alarm probabilities for RSS-based distance estimators

In this section, we adopt the single-path Gaussian model of Equation (2) and we treat the estimation of the user-to-user distance 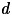 as a classical detection theory problem, where the hypotheses 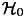 and 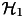 are defined as 3
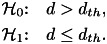
 It follows that the detection 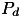, misdetection 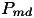 and false-alarm 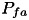 probabilities are defined as 4
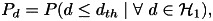

5
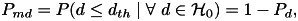

6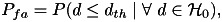 where 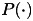 stands for conditional probability.

In what follows, we will focus only on 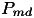 and 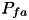, as the threshold choice (in terms of RSS observations) is a tradeoff between 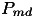 and 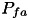, and as these two parameters reflect the false negatives and false positives in estimating the user-to-user distance below the safety threshold 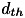.

By applying Bayes rules and using the RSS observations 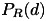, we arrive at 7
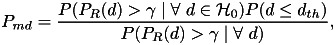

8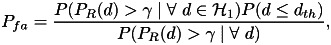 where 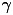 is the RSS threshold used to determine whether or not the user-to-user distance is below 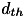. For example, if the receiver device measures from a BLE device in range an RSS higher than 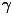 (e.g. 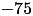 dBm), the receiver can conclude that the user-to-user distance is below the safety distance (e.g. below 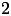 m).

Under the further assumption of users equally distributed in the considered space and assuming the path-loss model from Equation (2), we obtain after straightforward derivations that 9
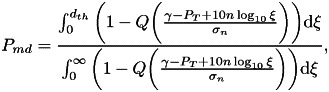

10
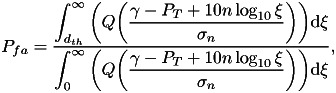
 where 

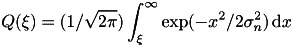

 is the well-known tail of a Gaussian distribution of zero mean and 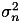 variance, i.e. the 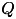-function. It should be noted that usually the RSS threshold 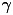 is set in such a way as to reach a certain target 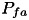 probability (e.g. 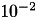 or 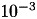). Examples of 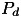 for different 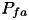 and path-loss models are provided in Section 5.3.

### 4.3 Modelling the connectivity and software reliability

Models for the probability for 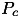 from Equation (1) depend on multiple parameters, such as the network, cloud and software reliability.

The network reliability, measured here as the probability 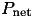 to have a wireless connection, depends on the wireless connectivity type used on the mobile device (e.g. cellular, low-power Internet of Things (IoT), Wi-Fi, etc.), as well as on the underlying physical layer of a certain connectivity solution, e.g. multi-carrier versus spread-spectrum, narrowband versus wideband, low-order versus high-order modulation, beam-forming versus no-beam-forming, etc.

The cloud reliability, measured here as the probability 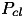 to communicate with the cloud server without errors, depends on the type of the network approach (e.g. centralised versus decentralised, opportunistic versus infrastructure-based, etc.) and possibly on the delay jitters and other synchronisation errors between the servers of a distributed cloud.

The software reliability, denoted here as 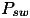 and representing the probability that the software app does not malfunction during a specific target duration, is a parameter dependent on the software creator, and various software-reliability models exist in the literature, such as the non-homogeneous Poisson process, Musa basic model, Musa–Okumoto model, Moranda model (Boland and Singh, 2003, see).

The probability 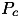 from Equation (1) can thus be written as
11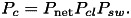 With the present wide spread of broadband connectivity in many countries and high-performance cloud services (e.g. Google Cloud, Amazon Web Services, Apple iCloud, etc.), it is fair to consider 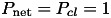. Therefore, 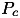 can be approximated by the software reliability 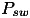. In what follows, we adopt the software reliability model of Boland and Singh (2003), which computes 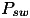 as a function of two modelling parameters: 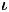, the rate of appearance of a fault per hour, and 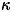, the probability of not fixing a fault within an hour. Besides these, 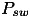 depends on the time interval of 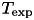, expressed in hours, and during which the app should be on and functioning correctly. For example, if an exposure notification will be triggered when a person stays in close proximity to an infectious person for at least 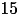 min, then 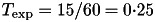 h.

According to Boland and Singh (2003), 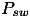 can be computed as 12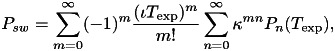 where 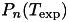 is the probability to have exactly 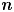 failures during the time interval 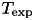, which is expressed as 13
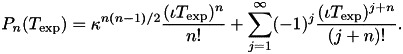


### 4.4 Modelling the infection risk

#### 4.4.1 Infection risk model without face masks

The infection risk model adopted here follows the epidemiological models by Buonanno et al. (2020a,2020b), where 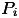, the probability of becoming infected after coming into contact with a person carrying SARS-CoV-2 virus, is modelled via 14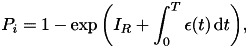 where 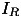 is the inhalation rate of the considered user, measured in m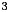/h, 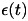 is the virus concentration (also called quanta) in an indoor environment at time 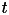, and 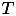 is the duration of exposure (measured in hours). Quanta concentration 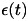, measured in quanta per volume (i.e. quanta/m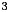) is given by 15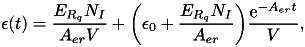 where 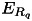 is the quanta emission rate of the infected person (measured in quanta/h), 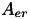 is the air exchange rate (in h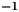) inside the considered space or volume, 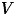 is the considered (indoor) volume, 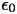 is a constant representing the quanta per volume at time 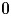 (in our simulations, we assume without loss of generality that 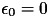), 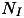 is the number of infectious persons in the considered volume, and 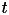 is the time. Examples of quanta emission rates are shown in Table 2, following the model by Buonanno et al. (2020a,2020b).

**Table 2.** Examples of quanta emission rates 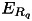 (quanta/h) for an infected subject with a viral load in the mouth 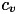 of 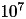 copies per millilitre

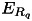 (quanta/h)	Resting	Standing	Light exercise
Silent/breathing	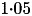	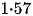	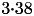
Voiced counting	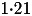	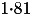	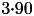
Speaking	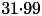	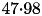	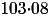

Equation (14) seems to be accurate for indoor spaces with limited ventilation. However, one can extrapolate this model to some extent and use it for certain outdoor areas. Of course, the extrapolation for outdoors, where quite a high level of ventilation is possible, may have some uncertainties associated with the results.

If we assume that the virus is spread while breathing (and talking, shouting or singing) by spherical droplets of diameter 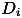, then the volume of each droplet is given by 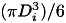 and the quanta emission rate 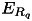 can be modelled as (Buonanno et al., 2020a)
16
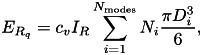
 where 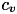 is the viral load in the sputum of the infectious person, measured in the number of ribonucleic acid (RNA) copies per millilitre of blood plasma, 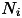 is the droplet concentration of the infected person (in particles per cm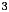) and 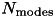 is the number of distribution channels during each expiratory activity by the infected person. Buonanno et al. (2020a) presented a model with 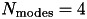, corresponding to four diameters of droplets, namely 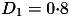 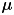
m, 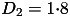 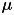
m, 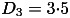 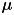
m and 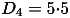 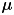
m, respectively. They also tabulated the average droplet concentration value 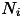 per mode, based on the work by Adams (1993). The inhalation rates 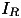 have also been modelled on average by Adams (1993) and Buonanno et al. (2020a,2020b) as a function of the activity of the infected person, namely in a resting position, in a static standing position or doing light exercise.

#### 4.4.2 Infection risk model with face masks

The infection probability in the presence of face masks can thus be theoretically modelled via Equation (17), by starting from Equation (14) with an additional factor 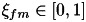 modelling the efficiency of the face-mask use. Thus, 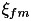 can range between the ideal 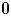 level (i.e. no additional protection through the use of face masks) to 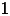 level (i.e. perfect protection ensured) 17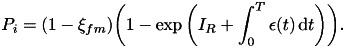 According to Liang et al. (2020) and based on a meta-analysis of 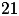 studies, 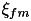 was found to range between 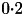 and 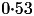, according to the type of face mask (e.g. surgical, FFP2, etc.), location (Western countries versus Eastern countries), scenario (healthcare versus non-healthcare workers) and face-mask adoption rate. Epidemiological mathematical modelling by Eikenberry et al. (2020) predicted a 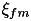 between 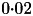 and 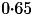 with moderately effective face masks and assuming up to 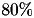 adoption of face masks within a population.

### 4.5 Modelling the user adoption rates

The user adoption rate 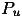 may depend on several parameters, including the perception of trustworthiness of the technology, the popularity of and adoption within the circle of social networks, the users’ digital and technological literacy and awareness, communication, and the transparency of the technology in terms of privacy, data management, authorities and access. A full list of the adopted applications worldwide may be found online (Tableau Public, 2020). Table 3 reflects the situation in February 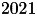 of ten selected contact-tracing apps worldwide, as examples of user adoption rates (implemented or forecast) since their launch on the market. We have calculated user adoption rates in relation to the whole population. However, some research shows that the adoption rate percentage could be estimated for specific population groups, such as tech-savvy or particular age categories.

**Table 3.** Statistics on the user adoption rates of the launched contact-tracing apps per country

Name of app	Target geographical region	Month of launch (in 2020)	Approximate adoption rate 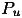 , in target region, as of February 2021
Apturi Covid	Latvia	May	N/A
Corona-Warn-App	Germany	June	22%
CovidTracker	Ireland	July	26%
Immuni	Italy	June	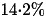
Koronavilkku	Finland	September	40%
NHS Covid19	United Kingdom (UK)	September	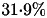
ProtectScotland	Scotland/UK	September	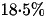
TousAntiCovid	France	October (relaunched)	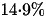
SwissCovid	Switzerland	May	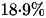
TraceTogether	Singapore	March	20%

**Figure 5.**
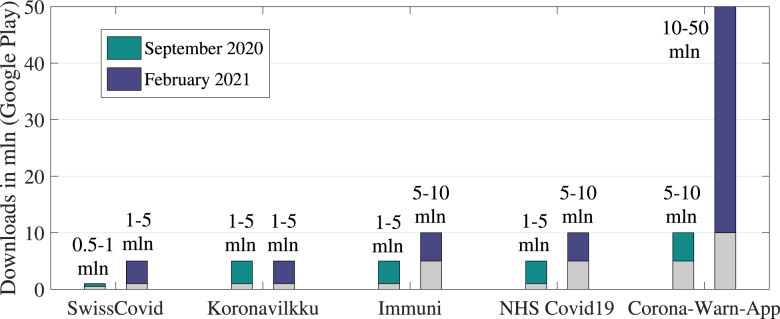
Statistics that indicate ranges on the app downloads, Google Play (in mln)

In order to assess the popularity of the apps among users, we selected five apps/countries and compared their download rates – as number of users expressed in millions (mln), as seen in Figure 5 – on Google Play: SwissCovid (Switzerland), Koronavilkku (Finland), Immuni (Italy), NHS Covid19 (United Kingdom) and Corona-Warn-App (Germany). The data as of September 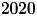 are published by Martin et al. (2020), and the data as of January 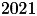 are available, according to the authors’ searches. The values available on Google Play are only available as intervals (e.g. between 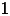 and 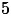 mln users), and not in absolute values. In addition, a parameter such as adoption rate depends entirely on such factors as the population level in the country and the number of active users (not only those who download an app). These relationships will be examined in more detail in Section 6.3, but, due to the different methods of how a user adoption probability can be computed, an exact comparison of the results in Figure 12 (Section 6.3) with those shown in Figure 5 here is hard to make.

Formulae to forecast the user adoption rates in the near future are hard to find in the current literature due to the multi-fold factors that may influence user adoption, such as governmental recommendations, the cost of an application (including the additional equipment cost if certain devices only support an app), the user trust in the service provider of a specific application, etc.

However, research on the minimum needed user adoption rates can be found. For example, in the study by Lambert (2020), a mathematical model was proposed to model the minimum adoption rate of a digital contact-tracing app necessary to curb an infection with epidemic reproduction number 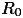, in such a way that 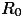 is brought below 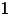 after a certain period of using of the digital contact-tracing app by the population.

Let us denote by 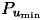 this minimum user adoption rate as per the Lambert (2020) model. It is assumed by Lambert (2020) that, if 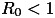, the epidemic curbs down (and disappears in time), by itself, without any additional digital contact-tracing methods. However, when the initial 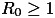, the 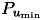 needed to curb the epidemic through a digital contact-tracing app was derived as
18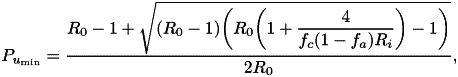 where 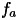 is the fraction of asymptomatic persons among the considered population, 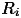 is the average number of secondary infections from symptomatic persons (given in number of persons) and 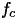 is the probability that a user cooperates and obeys the prevention orders, e.g. by following the recommended self-quarantine. Numerical examples of this minimum user adoption rate are further given in Section 6.

Forecasting models of user adoption rates of digital contact-tracing apps are still hard to find in the current literature. However, various diffusion models in the literature have been able to offer dynamic models of user adoption rates of various technologies (Orbach, 2016; Kapur et al., 2019; Liu et al., 2018). One of the most used technology-adoption diffusion models is the Bass model, according to Orbach (2016) and Kapur et al. (2019), describing the user adoption rate 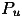 in time as 19
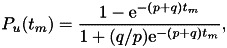
 where 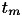 is the time expressed in months, and 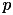 and 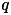 are two parameters of the model, defined as the fraction of early adopters 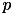 within a population, and the fraction of imitators or followers 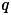 within a population. Clearly, 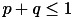, since some additional fraction 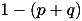 of the population can belong to another category of ‘indifferent adopters’. The meaning of an ‘early adopter’ in the above model is a person that adopts a new technology or software product very fast, and is not influenced by the number of persons that have already adopted such a product. An ‘imitator’ or ‘follower’ is a person that follows the trends and will adopt such technology only after such product/technology has been adopted by a high enough number of other persons, e.g. within their own circle of friends, family, colleagues, or social circle, etc.

In Section 6 (see Figure 12), a few examples will be given with varying 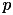 and 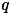 parameters that currently match the data from Table 3. While the Bass model is far from perfect in the context of a digital contact-tracing app, as it assumes that, once a technology or a product is adopted, it will remain on the market and there will be no drops in its usage, we believe that it can fairly predict short-term user adoption rates.

## 5. Measurement-based results for BLE RSS modelling

The following section provides an overview of RSS signal results by modelling the relation of the misdetection probability and the distance extracted from available measurements (the considered threshold for our assumptions stands at 2 m).

### 5.1 BLE calibration errors

Different mobile devices may report varying RSS ranges, according to Ma et al. (2017) and OpenTrace Calibration (2020), leading to errors if no calibration is performed. To perform calibration, offline measurements from various devices typically need to be collected beforehand at constant distances between transmitter and receiver. An open dataset for such calibration purposes was provided by OpenTrace Calibration (2020), based on measurements performed on 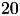 different Android and iPhone devices with 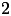 m distance between transmitter and receiver and BLE signals. Our analysis of the data by OpenTrace Calibration (2020) shows that the RSS mean over the considered devices at 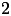 m is 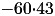 dBm, and its standard deviation over the considered devices is 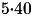 dB. This means that, in the absence of prior information about the device model and its calibrating parameters, the noise standard deviation of the path-loss modelling can increase by up to 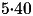 dB.

### 5.2 Example of a BLE path-loss model derived from measurements

**Figure 6.**
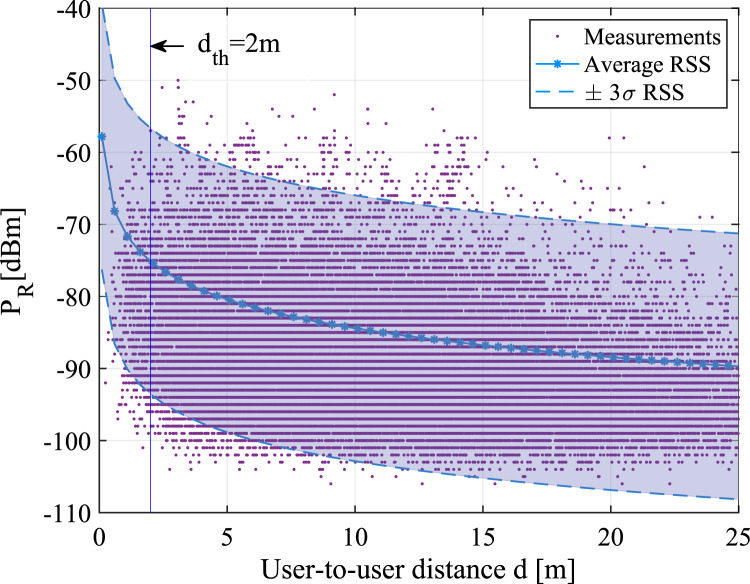
RSS versus distance according to the M4 indoor path-loss model from Table 1 with an example of 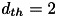 m safety distance threshold

Figure 6 shows an example of RSS fluctuations with distance, based on the BLE measurements done by Lohan et al. (2015) inside a four-floor university building that corresponds to model M4 from Table 1. The purple dots in Figure 6 show the instantaneous measurements, while the continuous blue line represents the average RSS based on the Gaussian single-slope model, and the shaded area stands for the 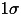 value. It should be recalled that, in a contact-tracing app, the measured instantaneous RSS is averaged over several minutes (e.g. the exposure duration is set to 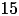 min typically). The averaged RSS over the exposure duration will converge to the average RSS value, even if instantaneous values have a wide spread, as seen in Figure 6. For clarity, we also provide the RSS 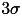 values as dashed lines in Figure 6.

### 5.3 Misdetection probabilities versus the safety distance threshold

**Figure 7.**
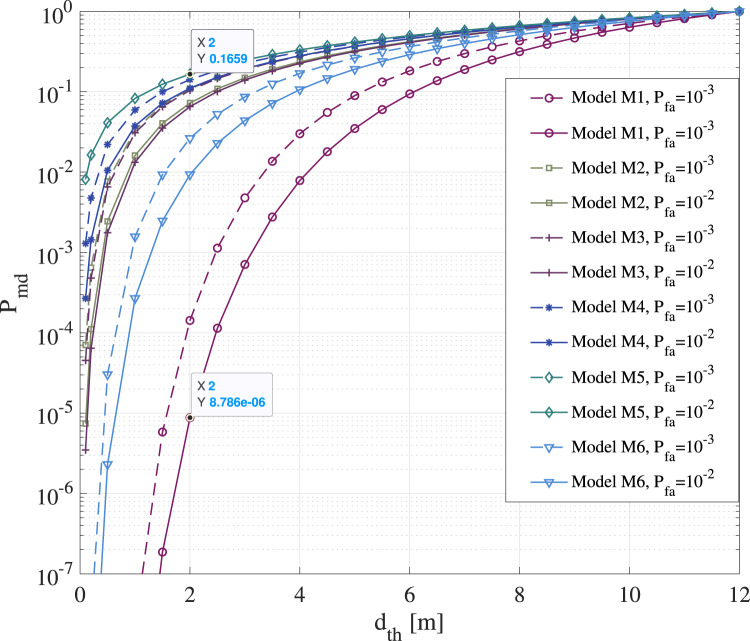
Misdetection probability versus threshold distance 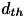 for six BLE path-loss models shown in Table 1 and derived from measurements

Figure 7 illustrates the misdetection probabilities 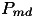 versus the chosen threshold distance 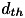 for six RSS channel models, namely M1–M6 from Table 1. Two fixed false-alarm levels (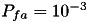 and 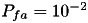) were used to compute the RSS threshold corresponding to a 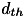 safety distance. Users were assumed to be uniformly distributed within a circle of maximum radius of 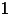 km, with a granularity of 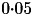 m. Results were obtained based on the theoretical models in Equations (9) and (10).

According to Figure 7, the RSS-based distance estimates can give misdetections with probabilities between 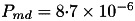 (model M1) and 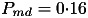 (model M5) for a typical 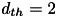 m distance, depending on the wireless channel propagation. Most of the considered models from Table 1 have misdetection probabilities larger than 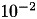 at 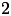 m threshold distance. As expected, the higher the RSS fluctuations are, i.e. the higher the shadowing variance 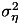 of the wireless channel is, the higher the misdetection probabilities are. As seen in Figure 7, increasing the safety distance threshold 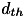 also increases the mid-detection probabilities. It means, for example, that, if the safety distance below which users can become infected if in contact with an infectious person increases to 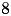 m, as shown for example in some recent studies by Bourouiba (2020), the digital contact-tracing app will have an increased number of misdetections and therefore lower effectiveness.

## 6. Simulation-based results based on our multidimensional modelling

The following section introduces the examples of the infection risk, software reliability and user adoption probabilities based on current rates. This section discusses the effectiveness of digital contact-tracing apps based on the two scenarios of wearing and not wearing face masks.

### 6.1 Illustrative examples of the infection risk

**Figure 8.**
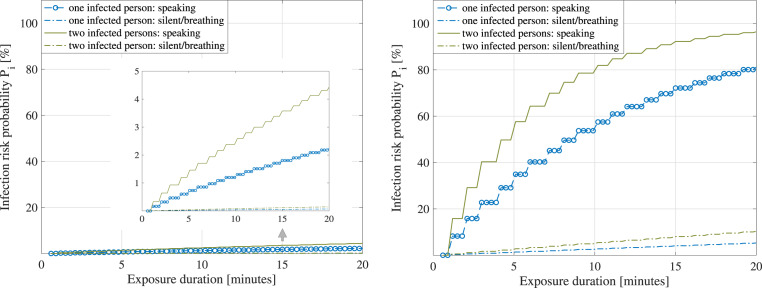
Large shopping mall. Left: Average infection risk 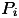 versus exposure times, 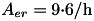 (mechanical ventilation). Right: Average infection risk versus exposure times, 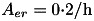 (natural ventilation)

**Figure 9.**
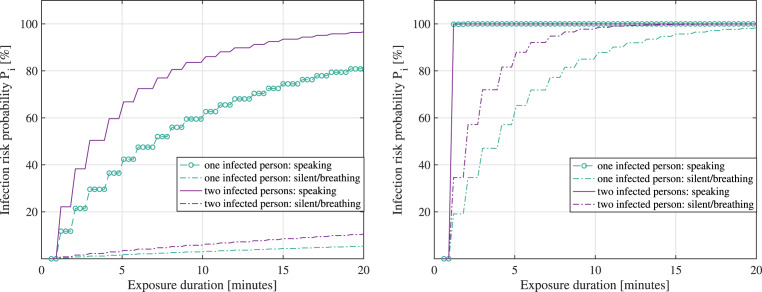
Small lecture room. Left: Average infection risk 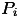 versus exposure times, 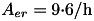 (mechanical ventilation). Right: Average infection risk versus exposure times, 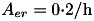 (natural ventilation)

Figures 8 and 9 follow the model in Equation (14) and show the probability to become infected versus the exposure duration, when in the vicinity of one or two infectious persons, in two scenarios: a large shopping mall/centre, of 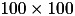 m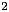 surface and 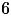 m height; and a small lecture room, of 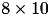 m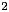 surface and 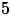 m height. For illustrative purposes, a scenario with manual ventilation and an air exchange rate 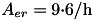 is shown on the left, and a scenario with natural ventilation and an air exchange rate 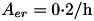 is shown on the right (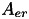 rates taken from Buonanno et al. (2020a)). Both silent and speaking cases of the infectious persons are considered, according to Table 2. The assumed viral load 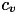 was moderate (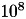 number of RNA viral copies per ml of blood, according to Buonanno et al. (2020a)). An inhalation rate 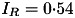 h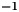 corresponding to a standing person was used according to values reported by Buonanno et al. (2020b).

It is to be remarked that the duration of exposure in the infection risk model of Equation (14) represents the amount of time spent in the same space (e.g. small office room or large shopping mall) with an infectious person, and it does not take into account the safety distance 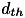 in the model. One can extrapolate that the model relying on Equation (14) and resulting in Figures 8 and 9 is thus more accurate for small-scale spaces than for large-scale spaces.

As seen in Figures 8 and 9, the infection risk increases as the exposure duration increases, when the number of infectious persons sharing the same physical space increases and when the size of the shared room decreases.

According to the adopted infection risk model given in Equation (14) as little as a few minutes in the vicinity of two infectious persons simultaneously speaking loudly in a small room can bring on a 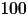
% infection probability, while a 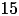 min exposure to two infectious persons simultaneously speaking loudly in a large shopping small with good manual ventilation brings in only about 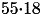
% infection risk. Owing to the fact that parameters such as the immunity system aspects were not parametrized in Equation (14), one can infer that estimated infection probabilities, as shown in Figures 8 and 9 are upper bounds or worst-case scenarios rather than average probabilities for assumptions from the scope of our study on multidimensional modelling on how to tackle the spread of the disease more efficiently.

### 6.2 Illustrative example of the reliability of mobile contact-tracing apps

**Figure 10.**
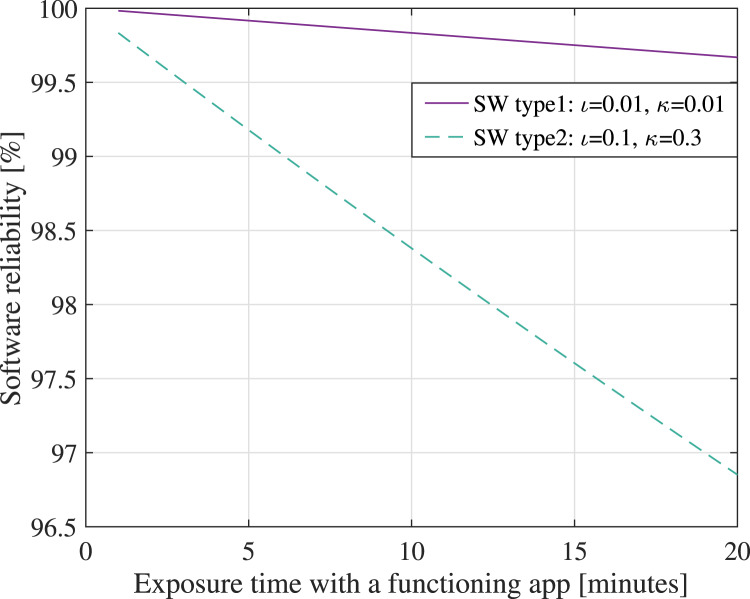
Software reliability 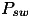 versus the exposure time with the app being active, assuming two software models: a high-reliability software (type 1, with a low rate of failures and fast failure fixing rates) and a lower-reliability software (type 2, with a higher rate of failures and lower failure fixing rates than type 1)

In this subsection, we discuss the circumstances that might affect the reliability of mobile contact-tracing apps and model this metric via high-reliability software and lower-reliability software assumptions and illustrate these via two examples. The results are shown in Figure 10. Clearly, the higher-reliability software (i.e. low rate of failures and fast failure fixing rates) has much higher 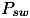 (close to 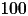
% than the lower-reliability software (i.e. with a high rate of failures and low failure fixing rates). The ratio between the rate of failures and the rate of fixing them thus influences the overall reliability 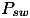 of the app. Finding realistic values for the rate of failures and failure fixing rate is not a trivial task and may depend on each software provider, as well as on each mobile device.

### 6.3 Illustrative examples of user adoption probabilities

**Figure 11.**
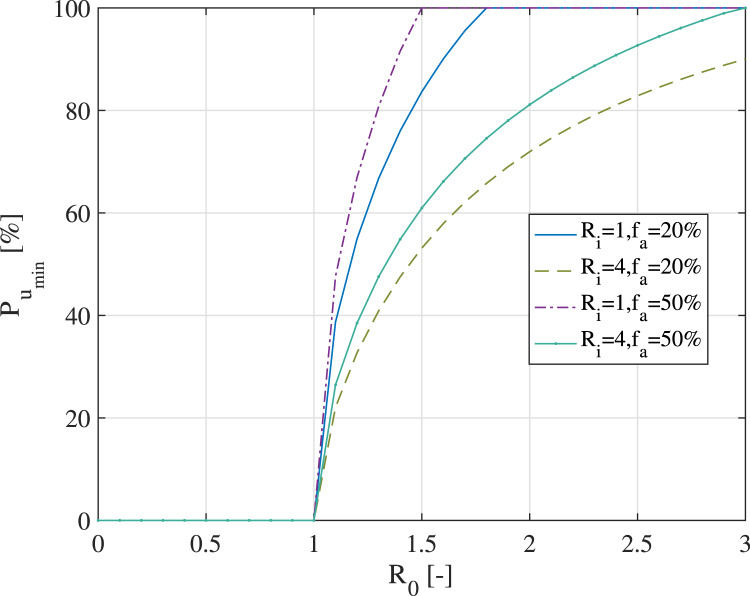
Minimum required user adoption rate for an effective digital contact-tracing app, according to the model by Lambert (2020)

Figure 11 shows the minimum required user adoption probability for a digital contact-tracing app, according to the model by Lambert (2020) and Equation (18). We assumed a 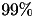 user cooperation probability (i.e. 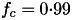), two levels of the percentage of asymptomatics within a population (namely 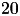
% and 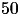
%), and two levels of the average number of secondary infections from symptomatics (namely 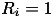 and 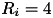).

According to Figure 11, a higher user adoption threshold is clearly needed when 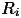 decreases, when 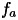 increases and when 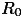 increases. It is to be recalled that the 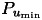 threshold was optimised with respect to no action relying on digital contact tracing; therefore, if the number of secondary infections 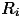 is small, the utility of a contact-tracing app comes only from wide adoption. Otherwise, there would not be many benefits over no digital action. Similarly, when the reproduction rate 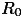 increases, a higher user adoption rate is needed to curb the curve of infections. Figure 11 also shows that it is impossible to give an absolute number for the needed minimal user adoption rate: under certain assumptions, even a 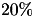 user adoption rate can bring in effective benefits compared to a no-digital-action approach; while under another set of assumptions (such as high 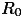, small 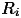 and high 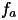), not even an 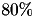 adoption rate may prove sufficient.

**Figure 12.**
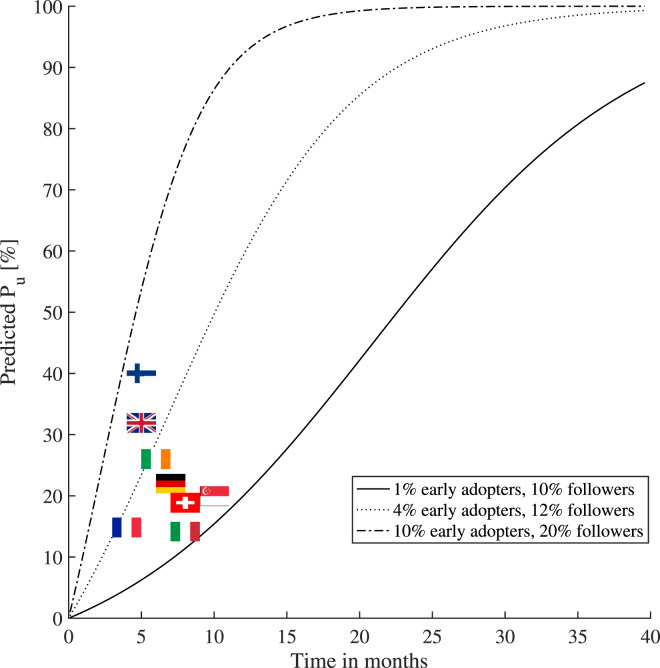
The variation of the contact-tracing apps user adoption rates per country (status as of February 2021) and the comparison with the Bass diffusion model under various parametric assumptions regarding the percentages of early adopters and followers within a population

Figure 12 shows the prediction of user adoption rates in time (expressed in months counted from the launch of an app), according to the Bass diffusion model, according to Orbach (2016) and Kapur et al. (2019). The realised user adoption rates, as reported in Table 3, are also plotted via the corresponding country flag for comparison purposes.

The percentages of early adopters and followers within a population shown in Figure 12 were drawn as illustrative examples to match some of the current user adoption figures. For example, assuming 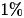 early adopters and 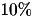 followers within a population (i.e. with the remaining 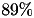 being indifferent to that particular app), one would get the lower-bound curve in Figure 12, which would match the current adoption rate in Italy and Switzerland (see Table 3). Also, assuming 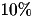 early adopters and 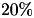 followers within a population, one would get the upper-bound curve in Figure 12, which would match the current adoption rate in Finland (see Table 3). The Bass diffusion model has its limitations when one uses it in the context of contact-tracing apps, as it assumes a continuous increase of the adoption rate in time and it does not model the end of a pandemic (and thus the end of the app's usefulness), or the users stopping the use of an app. Nevertheless, we believe that the Bass model is valuable to offer a coarse prediction of the short-term behaviour (e.g. within a few months to one year) regarding the adoption rates of a digital contact-tracing app.

**Figure 13.**
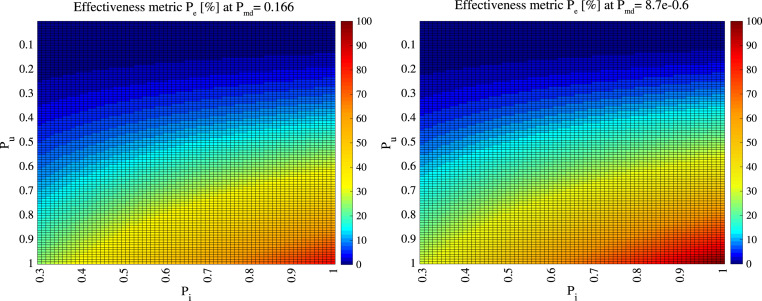
The effectiveness metric for a digital contact-tracing app at various 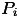 and 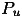 levels. Left: High misdetection probability 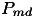 (e.g. corresponding to model M5 of Figure 7). Right: Low misdetection probability 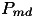 (e.g. corresponding to model M1 of Figure 7)

**Figure 14.**
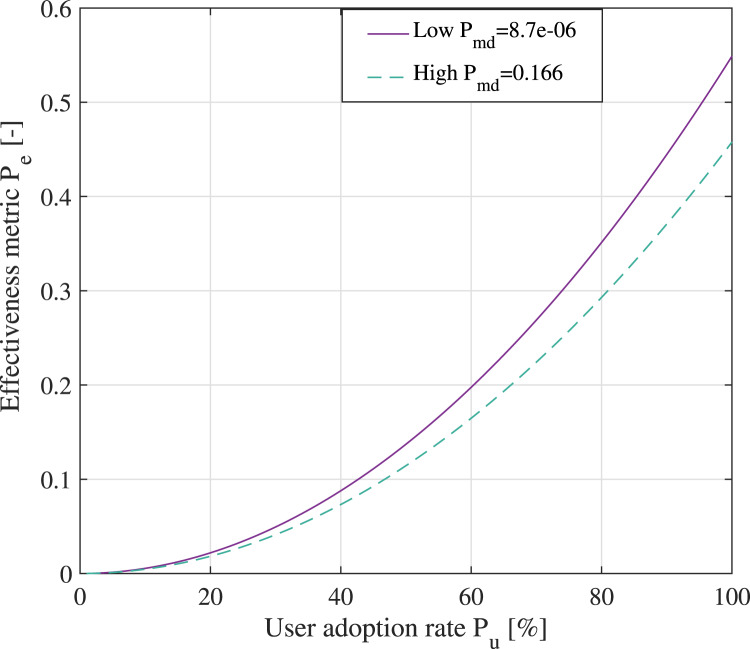
The effectiveness metric for a digital contact-tracing app under the assumption of 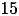 min exposure to two infectious persons in a large shopping mall

It is to be noted that, according to Briers et al. (2021) and based on the recently released data on the UK contact-tracing app adoption rate, their analysis showed that for every 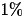 increase in the number of users of the app, the number of infections can be reduced by 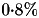 (from modelling) and by 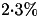 (from statistical analysis). Our results based on the presented model (and shown later in Figures 13 and 14) predict that, on average over all possible infection probabilities, or every 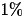 increase in the number of users of the app, the number of infections can be reduced by 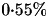 (i.e. slightly more pessimistic estimate than the one discussed in Briers et al. (2021)). If infection probabilities 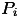 are assumed to be above 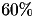, our model in Equation (1) also predicts a decrease of 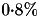 in the infections for every 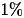 increase in the number of users of the app.

### 6.4 Illustrative examples of the effectiveness of digital contact-tracing apps

Figure 13 shows the effectiveness metric 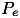 in percentages, according to various levels of infectiousness probability 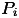 and user adoption rates 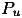 at two levels of a misdetection probabilities (a high one 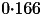 in the left-hand plot and a very low one of 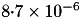 in the right-hand plot). Colours towards the red end of the spectrum show high effectiveness, while colours towards the blue end of the spectrum show low effectiveness compared to cases without digital contact-tracing solutions in use. One of the take-away messages from Figure 13 is that, while digital contact tracing is a promising disease control method and can significantly help in disease prevention under some assumptions (such as large 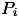 and large 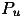), it is by no means able to mitigate the spread of the disease in a stand-alone mode completely and needs to be complemented by other preventive measures, such as physical distancing, good indoor ventilation, proper use of face masks, good hand hygiene, regular surface cleaning, fast testing and isolation procedures, etc.

Figure 14 provides examples of effectiveness metrics for a digital contact-tracing app versus its user adoption rate for low and high misdetection probabilities of the proximity detection algorithm. As seen in Figure 14, with a low user adoption rate of the digital app, there is very little difference between low- and high-misdetection-probability cases. With higher 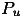 rates, a digital app with a very good proximity detection algorithm (i.e. low 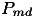) can be 5–8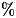 more effective than a digital app with coarse proximity detection estimates.

Table 4 specifies parameters used for modelling the two scenarios and provides the comparison for two different premises with multiple epidemiological assumptions.

**Table 4.** Estimation parameters for considered scenarios

Parameter	Scenario 1	Scenario 2
Voice type	Silent/breathing	Whispered counting
Activity type	Standing	Standing
Face-mask efficiency factor, 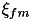	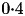	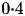
Indoor space type	Shopping mall	Lecture room
Indoor space volume (m 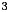 )	30,000	500
Number of infected persons	4	1
Exposure duration (min)	15	15
Fraction of adopters, 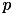	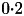	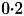
Fraction of followers, 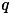	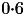	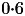
Viral load, 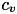 (RNA copies/ml)	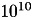	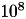
Ventilation rate, 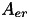	6	9
Rate of appearance of software fault, 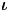	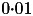	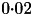
Probability of not fixing software fault, 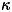	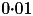	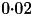
Misdetection probability, 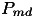	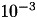	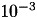

Finally, Figure 15 outlines the holistic model and shows examples of a digital contact-tracing app under two scenarios, with parameters listed in Table 4, and assuming both use of face masks and no use of face masks within the population. When face masks are used, a linear dependence on the user adoption rate 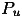 is assumed, with a face-mask efficiency coefficient 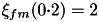
% at up to 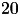
% user adoption rate and a face-mask efficiency coefficient 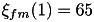
% at 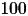
% user adoption rate (following the model in Equation (17) and the parameters described by Eikenberry et al. (2020)). The user adoption rate 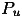 is assumed to increase in time, according to the Bass model from Equation (19), and the cloud and software reliability factors are also time-dependent. The infectious rate is computed based on parameters shown in Table 4, assuming 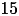 min exposures, either in a large shopping mall with four infectious persons or in a small lecture room with one infectious person. The maximum levels of 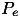 are highly dependent on the assumed scenarios, but the shape of the four curves shown in Figure 15 remains the same for all scenarios. Basically, the trend is that 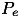 is constantly increasing until it reaches a saturation region, where 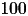
% users are using the digital contact-tracing app. When face masks are used within a population, the infectious rate 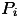 decreases, according to the model in Equation (17), and thus the effectiveness rate also decreases compared to the case where no face masks are in use.

**Figure 15.**
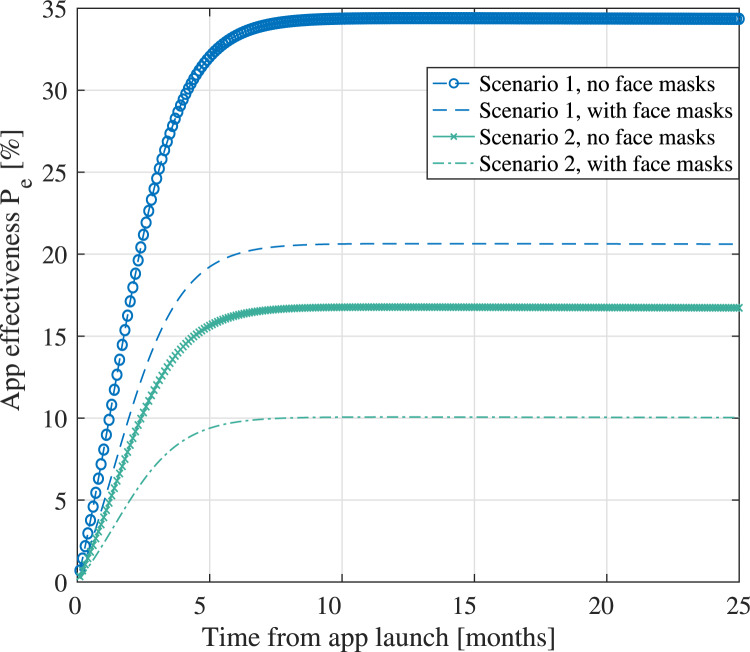
Predicted effectiveness of digital contact-tracing apps in time, under the two scenarios in Table 4 and derived from measurements

According to University of Zurich (2021), the effectiveness of SwissCovid, estimated as the number of correctly notified persons that were in contact with an infectious person and additionally were tested positive after an application alert, was computed as one in ten, namely around 10%, at seven months of the post-SwissCovid app launch. This estimate of approximately 10% effectiveness also matches our current models, e.g. as clearly seen in the lower curve of Figure 15 and blue areas of Figure 13.

## 7. A glance towards future perspectives and open challenges

**Figure 16.**
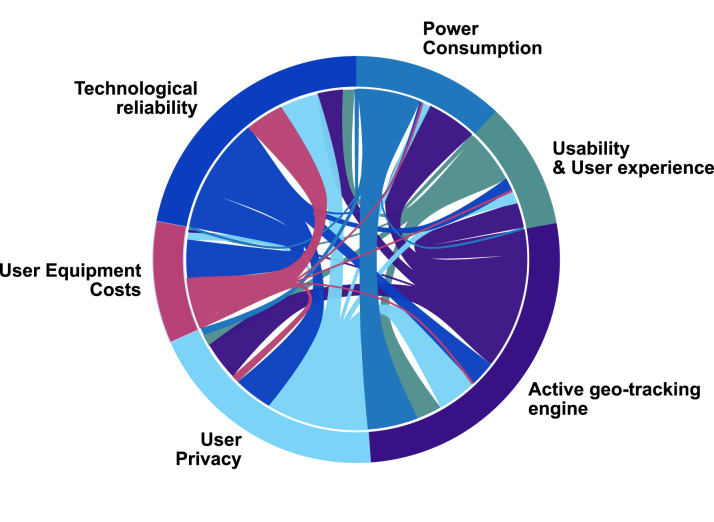
Design challenges and their interdependence towards wide adoption of a digital contact-tracing app

For the purpose of illustrating the multidimensional dependences between the different design parameters in a digital contact-tracing app, we provide Figure 16, presented as a talent–chord diagram. We consider the following characteristics to be interconnected and influenced by each other.

### 7.1 User privacy

As discussed in the survey by Shubina et al. (2020a), there are two decision-making approaches that allow digital contact tracing to be performed. Generally, the more the tasks are distributed within the system (i.e. no node having full information), the more privacy-preserving a solution is. Therefore, intuitively speaking, decentralised approaches should be able to provide better protection of the user's privacy than centralised approaches. However, to the best of the authors’ knowledge, there are currently no comprehensive studies available on whether centralised or decentralised approaches for contact tracing can preserve privacy better, and both approaches have similar vulnerabilities, as stated by Vaudenay (2020). According to Martin et al. (2020), each application has its own privacy policy on data processing, and this information is provided on the webpages of digital contact-tracing apps. Gvili (2020) provided a thorough security analysis that could shed some light on privacy tradeoffs in the GAEN approach. The study by Wen et al. (2020) evaluated privacy levels of different contact-tracing apps. The analysis of the extracted BLE property data with their semantics shows that several apps store invariable identifiers that allow tracing paths of a specific user. Therefore, one could conclude that privacy issues affect the user adoption rate of the application directly and indirectly.

### 7.2 Power consumption

Some mobile device features, such as the display components, processor and network connectivity, can be considered power-hungry, depending on the frequency and the device in use. In scenarios where citizens use a digital contact-tracing app continuously, power consumption appears to be a serious concern. Power consumption on the user's device is highly correlated with the frequency of scanning or scanning intervals. Leith and Farrell (2020a) showed that a widely considered GAEN protocol implementation only scans the nearby beacons every 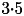
–5 min as a tradeoff between accuracy and power consumption. However, such a large scanning interval may be insufficient to attain accurate detection probabilities and low false-alarm rates. Therefore, finding the appropriate balance between battery consumption and frequency of measurements remains a critical research subject. Moreover, as per Ahmed et al. (2020), data fusion for improving proximity accuracy, technologies such as BLE, UWB, and other sensor data potentially available on smartphones, e.g. GPS, gyroscopes, accelerometers and magnetometers, could increase battery consumption even more on the user's device.

### 7.3 Usability and user experience

Usability as a characteristic includes ease of use and user trust in the app. The study by Simko et al. (2020) focuses on investigating the values and concerns of society regarding apps for digital contact tracing. The survey results indicated that at least 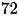
% of participants would probably download an app which would protect their data and would not disclose such data to third parties.

### 7.4 Active geo-tracking engine

Continuously active positioning techniques on mobile devices might possibly cause battery drainage, depending on the technical characteristics of the device. However, this feature is an essential component for enabling location-based services, according to Basiri et al. (2017). There are a number of wireless technologies that can be used for positioning, such as Wi-Fi, BLE, RFID and UWB, among others.

### 7.5 User equipment costs

The devices, applications and connectivity might vary significantly on different devices. Moreover, not all users possess mobile phones with a stable connection to the cloud. Therefore, in order to perform efficient digital contact tracing, users might need to have a device or a tag provided by third parties.

### 7.6 Technological reliability

The accuracy of estimates is highly dependent on cloud reliability and the exposure notification system's reliability. As seen so far, the use of BLE for social distancing has gained a great deal of attention in the context of digital contact-tracing apps; however, there remain several technical limitations that need to be addressed in order to increase the reliability and accuracy of proximity sensing for such a sensitive application, i.e. social distancing at the time of COVID-19. According to findings by Basiri (2020), particular limitations still to overcome include the following:

- *Sensitivity and high noise of BLE signals.* Owing to their design properties, RSS is sensitive to wireless signal fluctuations, as for any RSS-based technology, due to the body and hand-grip orientation, presence of obstacles in the signal path, and other channel and environment dynamics.
- *High false-positive cases in the detection of transmission.* Obstacles, such as thin obstructions, may not significantly affect RSS to claim a high detection rate. Some barriers can stop the spread of the virus even though the distance is below the 2 m threshold, known as an epidemically safe distance. Nevertheless, to achieve high efficiency, a digital contact-tracing solution should perform obstacle detection: whether another person or a wall appears to be between two devices, within different proximity distances, such as 1 or 2 or 3 m.

Research groups from many countries are working on developing software based on existing examples. In particular, DP-3T serves as a framework for an entirely scalable application. There is currently no single solution for tackling the spread of COVID-19, for several reasons (the number of active users, privacy issues, energy consumption constraints, among others), and the restrictions mentioned earlier in the simulation. Another vital characteristic of the discussion is the method of obtaining users’ locations. In centralised approaches, multiple third parties are involved. Moreover, some applications use precise GPS coordinates, which raises significant ethical concerns.

The vast majority of the existing digital contact-tracing solutions have confirmed their effectiveness as a complementary step along with other precautionary activities that aim to tackle the spread of the virus. As can be seen, scientists may apply their efforts worldwide to solve a multidimensional issue to obtain better results in contact tracing, control and reduction of the spread of the contagious virus to stop the ongoing pandemic.

## 8. Conclusions

This work has evaluated digital contact-tracing effectiveness, used on mobile devices for tackling the spread of COVID-19, by assessing several aspects. By estimating the maximum prevention probability, we have obtained an upper bound of the effectiveness of a digital contact-tracing solution. This effectiveness parameter conveys the extent to which a digital contact-tracing app could help compared with the opposite situation. Particular factors, such as the type of wireless technology used for identifying the proximity between users, the availability of cloud connectivity and exposure duration, influence the efficacy of digital contact-tracing solutions.

An open research direction lies within the study of different proximity-detection approaches based on positioning techniques other than BLE, such as Wi-Fi, UWB or hybrid approaches, as this would enable better coverage and obtain better effects in digital contact tracing.

Besides the above-mentioned issues, ethical and privacy-related features play a vital role in the broad implementation of contact-tracing apps; therefore, these could be potential directions for future research. Multiple ethical constraints are currently related to the type of approaches chosen for contact tracing, including other medical and political matters.

## APPENDIX: Overview of digital contact-tracing apps

This appendix aims to provide a brief outline of existing technological solutions developed for tackling the spread of COVID-19 worldwide since the beginning of 2020. We conducted the comparison among the selected  contact-tracing apps and protocols in Table A1). The main characteristics are the IoT connectivity type, proximity detection methods and the standard of approach. Furthermore, we analysed qualitative preferences concerning their energy efficiency, accuracy and privacy levels, according to literature reports. The current prevalence of these  apps in technical repositories and their variability in the contact-tracing methods deployed is also considered. To ensure that a broad review of reasonable digital solutions is presented, we note that nowadays there are at least  mobile applications in at least  countries worldwide (Tableau Public, 2020), in addition to the applications overviewed in Table A1. At the first step, the vast majority of mobile apps for contact tracing approved by governments had proprietary features and relied on centralised data processing. However, with technological advances, most solutions rely on the GAEN framework, which assumes a decentralised approach for data collection and processing. The most popular technology is BLE and RSS measurements, which claim to be a practical choice due to their wide spread.

**Table A1.** Qualitative comparison of existing COVID-19 contact-tracing protocols in use

Protocol/	Wireless	Proximity		Detection	Energy		Main advantages (A)
reference	technology	detection	Architecture	accuracy	efficiency	Privacy	Specifics (S) App examples (E)
DP-3T/Troncoso et al. (2020)	BLE	RSS	DC				**A:** Privacy-by-design preserved; open-source protocol; no user location is stored in any parts of the tracing chain. **S:** High variability of BLE RSS measurements make precise distance estimates difficult; to fully preserve privacy, it also requires manual and timely user feedback. **E:** SwissCovid
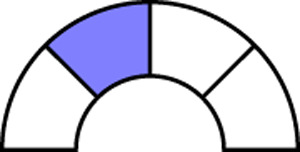
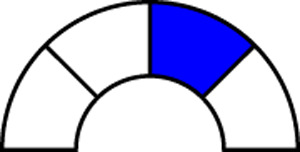
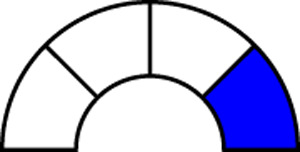
TCN/TCN Protocol (2020)	BLE	RSS	DC				**A:** A decentralised protocol that provides interoperability to contact-tracing apps. **S:** Also high variability of BLE RSS; claims to function without the healthcare service provider involved. **E:** Covid Watch, CoEpi
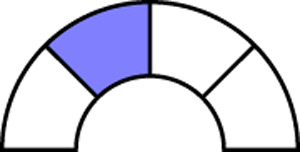
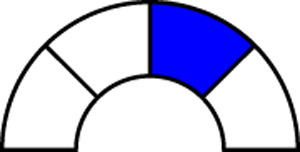
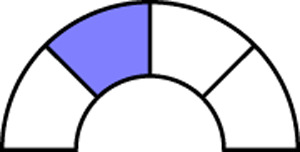
EPIC/Altuwaiyan et al. (2018)	BLE and Wi-Fi	RSS	C				**A:** Encryption offers certain privacy protection to the centralised approach; combined Wi-Fi/BLE decreases the misdetection probability. **S:** Central server may be prone to malevolent attacks; RSS results reported by heterogeneous devices need to be calibrated.
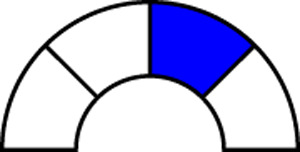
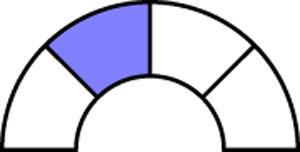
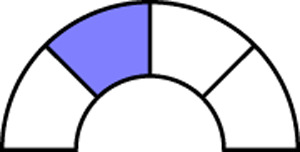
GAEN/Michael and Abbas (2020)	BLE	RSS	DC				**A:** Privacy-by-design preserved; might become a default option; bigger market share than for other apps. **S:** High variability of BLE RSS measurements make precise distance estimates/exposure detection estimates challenging. **E:** More than apps already deployed worldwide
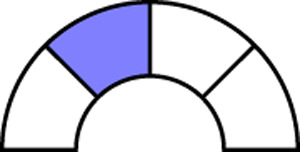
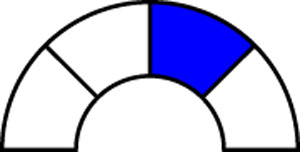
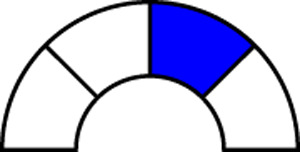
PEPP-PT/PEPP-PT (2020)	BLE	RSS	C				**A:** Centralised solutions are more scalable than decentralised ones. **S:** Lower privacy than in decentralised approaches might hinder mass adoption.
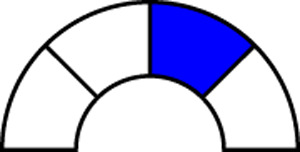
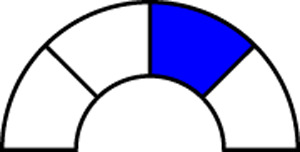
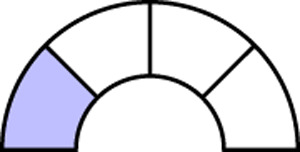
ROBERT/Cunche et al. (2020)	BLE	RSS	DC				**A:** Open-source protocol, highly scalable. **S:** It has some restrictions due to minimum requirements of the operational system of the device. **E:** StopCovid (France)
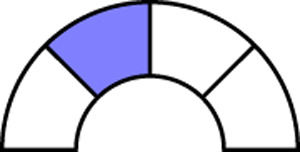
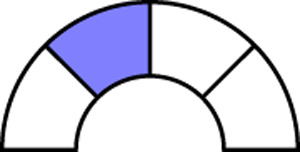
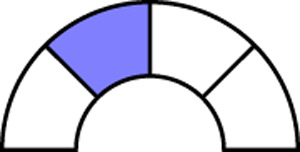

The solutions are presented in Tables A1 and A2. The first one in Table A1, namely the Decentralised Privacy-Preserving Proximity Tracing or DP-3T (Troncoso et al., 2020), is a BLE-based decentralised protocol for contact tracing, currently available on the Swiss market and in a few other EU countries. The DP-3T protocol also served as an example for the Google/Apple (GAEN) protocol, and it contends to be one of the protocols preserving best the user's privacy when a contact-tracing app is used. Here, the proximity estimates between two devices rely on RSS; therefore, they are susceptible to wireless signal fluctuations, as any RSS-based technology, due to the body and hand-grip orientation, the presence of obstacles in the signal path, and other channel and environment dynamics.

**Table A2.** Qualitative comparison of existing COVID-19 contact-tracing solutions in incipient or research stage.

Application	IoT	Proximity		Detection	Energy		Main advantages
reference	solution	detection	Architecture	accuracy	efficiency	Privacy	(A) Specifics (S)
MIT SafePaths/MIT (2020)	BLE, GPS	RSS, AOA and TOA	DC				**A:** Privacy-by-design; the use of GPS decreases the misdetection and false-alarm probabilities. **S:** Privacy might be adversely affected as some parties (e.g. health unit) may get access to GPS tracks.
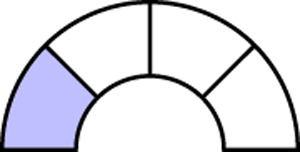
Research Work/Nguyen et al. (2020c)	BLE and Wi-Fi	RSS, magnetic field/sound intensities	C				**A:** May cope better with wireless signal variability than BLE alone, as multiple sensors are hybridized. **S:** User privacy hard to preserve; devices need to be equipped with many sensors; high energy consumption.
MokoSmart/ MokoSmart (2020)	BLE, LoRa	RSS	C				**A:** Already tested with wearable devices; can provide proximity alerts if the distance is less than m. **S:** Centralised servers are prone to privacy breaches/ malevolent attacks.
LocalSense/Tsingoal/ Tsingoal (2021)	UWB	RTT	C				**A:** Very accurate distance estimates; channel variability (such as shadowing) has low impact. **S:** Need for additional infrastructure (e.g. UWB gateways); low-to-medium energy efficiency.
TraceTogether/Abbas and Michael (2020)	BLE	RSS	C				**A:** The app and physical tokens as parts of a systematic approach are already adopted by the Singapore government. **S:** Server robustness must be at the high level.

C – centralised approach DC – decentralised approach

– low  – medium

– high  – very high

The TCN Protocol (TCN Protocol, 2020) is a solution used for proximity detection, also based on BLE RSS distance estimates to detect nearby users.

Another RSS-based solution, this time with a centralised server storing encrypted user information and computing a score matching calculation to detect if users have possibly been infected by a close COVID-19 positive contact, is the EPIC protocol, as described by Altuwaiyan et al. (2018).

The Google/Apple (GAEN) protocol, as discussed by Michael and Abbas (2020), also relies on BLE RSS-based distance measurements between devices and a decentralised approach similar to DP-3T. No location information needs to be stored about the users; however, Google location services activate both BLE and GPS engines. The main difference with DP-3T is that the DP-3T is a fully open-source protocol, while some parts in the Google/Apple app will remain proprietary.

The Pan-European Privacy-Preserving protocol, PEPP-PT (PEPP-PT, 2020), is a centralised proximity-tracing application that relies on measuring BLE radio signals. According to the specification, the protocol logs the beacons’ encrypted history in proximity in scenarios where two devices are located closer than the ‘safe’ distance for a reasonable time. The user devices store each other's anonymous identifiers exclusively. Consequently, no sensitive personal information, such as geographic location, is collected. More past events are deleted after some time to follow the data minimisation principle.

Arguing that there is no fully decentralised approach available today, Cunche et al. (2020) proposed a ROBust and privacy-presERving proximity Tracing (ROBERT) protocol, which is built on federated server infrastructure and uses temporary anonymous identifiers.

While Table A1 focused on more established and already deployed protocols and apps, Table A2 summarises some more experimental solutions, in order to offer a broad view of potential technologies and solutions for contact tracing.

The MIT (2020) SafePath application from Table A2 is another open-source protocol with a decentralised decision-making process, which combines BLE RSS-based distance estimates between users with GPS-based own location trails. The fact that user GPS locations are also stored on their own mobile devices and sent to the cloud server in case infection occurs makes this solution less private than the DP-3T approach.

The solution described by Nguyen et al. (2020c) relies on multiple sensors available on a smartphone, such as BLE, Wi-Fi, magnetometers and sound sensors, and, through multi-sensor hybridisation, it could offer high protection against the wireless signal variability at the expense of a higher computational cost of integrating heterogeneous measurements from multiple sensors.

MokoSmart (MokoSmart, 2020) is a wearable device developed by a Chinese company for COVID-19 contact tracing and integrates BLE and LoRa technologies in a centralised approach. Another well-defined wireless connectivity approach is offered by the Tsingoal company (Tsingoal, 2021), through their LocalSense system. The LocalSense approach is distinct from most other contact-tracing solutions in being based on UWB and round-trip time (RTT) measurements. The enhanced accuracy of the distance estimates between users is the principal advantage of such a system. Generally, UWB RTT measurements are known to offer down to  m accuracy, particularly up to ten times more accuracy than BLE RSS solutions. The main drawbacks are the requirement for embedded UWB chipsets (at the user side), infrastructure and the potential privacy breaches due to centralised data storage.

TraceTogether is a different case of a proximity-tracing solution that relies on BLE signals (Abbas and Michael, 2020). As a plausible limitation, this application should be running in the mobile phone background continuously. This requirement might cause the battery drain of the device. Through encrypting the user ID, anonymous identifiers are generated with a private key and are shared solely with governmental-approved organisations.

Available COVID-19 digital contact-tracing solutions, applications and/or protocols can be characterised with diverse metrics, among which the following three are very important in the authors’ opinion: the detection accuracy at different levels of user-to-user proximity, energy efficiency and privacy. These three performance metrics are compared qualitatively among the  solutions from Tables A1 and A2.

Typically, there is a tradeoff between the accuracy level (in relation to proximity detection) and the level of achievable privacy (high accuracy levels can be typically achieved with a decreased user data privacy and vice versa). Following this flow of thought, a pattern is shown regarding decentralised applications that typically fare better than centralised solutions in the context of privacy (due to distributed data processing), but may be limited in terms of achievable accuracy bounds.
